# Quantifying habitat impacts of natural gas infrastructure to facilitate biodiversity offsetting

**DOI:** 10.1002/ece3.884

**Published:** 2013-12-12

**Authors:** Isabel L Jones, Joseph W Bull, Eleanor J Milner-Gulland, Alexander V Esipov, Kenwyn B Suttle

**Affiliations:** 1 Department of Life Sciences, Imperial College London, Silwood Park CampusAscot, U. K; 2 Institute of Zoology, Academy of Sciences of the Republic of UzbekistanTashkent, Uzbekistan; 3 Department of Life Sciences & Grantham Institute for Climate Change, Imperial College London, Silwood Park CampusAscot, U. K

**Keywords:** Compensation, ecological impact assessment, residual impact, semi-arid, Uzbekistan

## Abstract

Habitat degradation through anthropogenic development is a key driver of biodiversity loss. One way to compensate losses is “biodiversity offsetting” (wherein biodiversity impacted is “replaced” through restoration elsewhere). A challenge in implementing offsets, which has received scant attention in the literature, is the accurate determination of residual biodiversity losses. We explore this challenge for offsetting gas extraction in the Ustyurt Plateau, Uzbekistan. Our goal was to determine the landscape extent of habitat impacts, particularly how the footprint of “linear” infrastructure (i.e. roads, pipelines), often disregarded in compensation calculations, compares with “hub” infrastructure (i.e. extraction facilities). We measured vegetation cover and plant species richness using the line-intercept method, along transects running from infrastructure/control sites outward for 500 m, accounting for wind direction to identify dust deposition impacts. Findings from 24 transects were extrapolated to the broader plateau by mapping total landscape infrastructure network using GPS data and satellite imagery. Vegetation cover and species richness were significantly lower at development sites than controls. These differences disappeared within 25 m of the edge of the area physically occupied by infrastructure. The current habitat footprint of gas infrastructure is 220 ± 19 km^2^ across the Ustyurt (total ∼ 100,000 km^2^), 37 ± 6% of which is linear infrastructure. Vegetation impacts diminish rapidly with increasing distance from infrastructure, and localized dust deposition does not conspicuously extend the disturbance footprint. Habitat losses from gas extraction infrastructure cover 0.2% of the study area, but this reflects directly eliminated vegetation only. Impacts upon fauna pose a more difficult determination, as these require accounting for behavioral and demographic responses to disturbance by elusive mammals, including threatened species. This study demonstrates that impacts of linear infrastructure in regions such as the Ustyurt should be accounted for not just with respect to development sites but also associated transportation and delivery routes.

## Introduction

Land-use change is a key driver of biodiversity decline through impacts on habitat availability (Mace et al. 2005). The extractive hydrocarbon, metal and mineral industries are among the most locally damaging forms of anthropogenic disturbance in many areas (Baillie et al. 2004). The negative impacts of extractive activities could be at least partially compensated for through “biodiversity offsets,” which endeavor to ensure “no net loss” of biodiversity alongside development by compensating for unavoidable ecological losses (ten Kate et al. 2004; Bull et al. 2013a). However, formal and comprehensive quantification of the landscape impacts of infrastructure in the relevant environment will be essential if the potential of offsetting is to be realized (Quintero and Mathur 2011). Accurate quantification of the residual impacts from development, which biodiversity offsets aim to compensate for, is often overlooked in the offset literature. Semiarid habitats may be particularly vulnerable to disturbance from industrial activities (Lovich and Bainbridge 1999), recovering poorly if at all (Fiori and Martin 2003) and with full suites of constituent species persisting only in remnant undisturbed patches (Rapport and Whitford 1999). Given that dryland (i.e. arid and semi-arid) biomes cover approximately 41% of the world's land surface and support over 38% of the global human population (Millennium Ecosystem Assessment 2005), it is crucial that responses to industrial disturbance in these habitats are better understood and managed.

In semiarid environments, habitats heavily disturbed by anthropogenic activities often have lower species richness compared with undisturbed areas (Simmers and Galatowitsch 2010). Even low-intensity and small-scale disturbances can have immediate and persistent effects (Forbes et al. 2001), with slow recovery times (Cui et al. 2009). The impacts of infrastructure upon vegetation in a variety of habitats – and of roads in particular, which are ubiquitous in regions of industrial extractive activity – have been well studied (Forman and Alexander 1998; Trombulak and Frissell 2000; Coffin 2007). Irrespective of the sector that uses them, roads can impact ecosystems in numerous ways: via non-native species brought in by vehicles (Gelbard and Belnap 2003); nitrous oxide and other pollutants produced by vehicles (Gadsdon and Power 2009); and by subdividing populations and forming physical barriers to dispersal that alter demographics (Forman and Alexander 1998). Thus, species richness (Lee et al. 2012) and total vegetation cover (Fiori and Martin 2003) may decrease in response to road construction. The magnitude of these effects will vary with distance from the actual disturbance. For example, in California, Gelbard and Harrison (2003) found that plant cover was significantly lower within 10 m of roads and that native species richness was impacted even 1 km away. Conversely, roads may actually enhance overall species richness (Zeng et al. 2011), for example, if associated disturbances provide favorable microsites for vegetation establishment (Boeken and Shachak 1994; Brown and Schoknecht 2001).

Natural resource extraction has had well-documented impacts on wildlife as well as vegetation (E&P Forum/UNEP 1997; Epstein and Selber 2002; Kumpula et al. 2011; OGP/IPIECA 2011). Habitat disturbance from industrial infrastructure can affect wildlife both spatially and temporally, for instance altering breeding patterns of birds (Walker et al. 2007) and grazing patterns of herbivores, leading to increased usage and pressure on surrounding undisturbed habitats (Vistnes and Nellemann 2007). Noise associated with transport and other activities along infrastructure may disrupt the use of habitat by faunal species (Rabanal et al. 2010), and the physical obstruction caused by pipelines can alter animal movement across landscapes (Curatolo and Murphy 1986; Dyer et al. 2002). In this manner, the spatial impacts of disturbance on the mean species abundance of vertebrate mammals in a range of habitats have been assessed to extend up to 5 km from infrastructure (Benítez-López et al. 2010). Vegetation responses may likewise be important to wildlife, altering resource availability and habitat structure. Further indirect effects may come from increased human use of the area that follows development, including water extraction, natural resource use, and hunting (Thibault and Blaney 2003). Finally, as with roads, industrial activity can be associated with the spread of invasive alien species (E&P Forum/UNEP 1997), although this has not been a conspicuous problem for the case study region (J. Bull, pers. obs.).

Many of the ecological impacts from industry can, in principle, be mitigated through biodiversity offsetting (“offsets”), but offsets are beset with implementation challenges, including the difficulty of fully quantifying biodiversity losses (Bull et al. 2013a). Quantification of the scale of disturbance impacts for offset projects often focuses on the impacts of development sites or “hubs,” whereas the disturbance from the construction and operation of linear infrastructure such as roads that link “hubs” is only recently being treated as something that could also be compensated for through offset mechanisms (Quintero and Mathur 2011). Further, if linear infrastructure has a comparable impact by area to hub infrastructure across a landscape, which to the authors' knowledge has not before been explored for offsets, then the scale of offsets required to achieve “no net loss” of biodiversity could be greater than currently thought.

### Biodiversity and natural gas extraction in semiarid Uzbekistan

Central Asian countries are predominately semi-arid and constitute an important proportion of the global semiarid biome, 16% of which is found in Asia. In Uzbekistan, over 99% of the country by area is arid and semi-arid (White and Nackoney 2003). Uzbekistan contains an estimated 27,000 species (USAID 2001; UNDP 2010). The transboundary Ustyurt Plateau, which Uzbekistan shares with Kazakhstan, covers ∼ 100,000 km^2^ of Uzbekistan and contains 271 recorded vascular plant species (Gintzburger et al. 2011), several of which are on the IUCN Red List (Esipov and Shomurodov 2011). The Ustyurt is also home to vertebrate species of high conservation priority, most notably the critically endangered saiga antelope *Saiga tatarica tatarica* (Mallon 2008). The saiga is the flagship conservation target for the Ustyurt, and its distribution and abundance are driven by both vegetation cover (Singh et al. 2010a) and sensitivity to anthropogenic presence (Singh et al. 2010b).

The Ustyurt is not only valuable from an ecological perspective, but also for the subterranean resources it contains. Uzbekistan is among the top 20 global exporters of natural gas (Effimoff 2000; EIA 2012), and this industry is expanding (Dorian 2006). Karakalpakstan, the administrative region containing the Uzbekistan part of the Ustyurt, is one of the country's main growth areas for gas extraction, with more than 3bn USD of foreign investment currently agreed for exploration and extraction activities (UNDP 2010; EIA 2012). Development of the Ustyurt for hydrocarbon production since the Soviet era has resulted in extensive infrastructure growth, including exploration and extraction sites, pipelines, and a substantial network of roads (UNDP 2010). To date, as far as we are aware, there has been no quantitative evaluation of the impact of roads, or other components of natural gas infrastructure (pipelines, extractive facilities), upon biodiversity on the Ustyurt.

### Quantifying disturbance on the Ustyurt Plateau

There are currently plans to design an offset policy in the Uzbekistan Ustyurt, to compensate for the ecological losses from development and conserve the regional saiga population, as part of a broader biodiversity conservation initiative for the Uzbek oil and gas sector (UNDP 2010; Bull et al. 2013b). Offset projects on the Ustyurt could be improved by the provision of sound scientific knowledge of the impacts of natural gas activity at the landscape level, including whether linear infrastructure impacts are important enough to necessitate inclusion in offset calculations. The types of impact we explore here – direct impacts on vegetation from industrial infrastructure, in an area of general conservation concern but without legal protection – could be considered to be unavoidable, residual impacts of the natural gas industry. These direct impacts are therefore appropriate for biodiversity offsetting (Quintero and Mathur 2011), which is an idea at the forefront of considerations for conservation in the Ustyurt (Bull et al. 2013b). Condition and area-based assessments of vegetation losses and gains form the basis of a number of existing offset policies (Quétier and Lavorel 2011), hence our focus on measuring impacts on vegetation quality (condition) and cover (area). We aim not only to quantify the local impacts of infrastructure on Ustyurt habitat, but also the landscape-scale habitat footprint of the oil and gas sector in a way that is relevant for the development of an appropriate biodiversity offset policy.

On the Ustyurt, the vast majority of roads created and used by the oil and gas industry are unpaved and formed from repeated vehicle travel rather than formal clearing and construction. Most roads are therefore temporary or seasonal, although arterial routes between established extraction facilities are large and effectively permanent. In addition to damage from direct impaction and clearing by vehicles, dust deposition could further impact plateau vegetation, as has been suggested by observations of dust cloud movement and settling following vehicle passage (Gintzburger et al. 2011). Dust can affect key physiological processes such as photosynthesis, respiration, and transpiration (Farmer 1993). In arid environments, dust can coat leaf surfaces, altering their radiation balance (Grantz et al. 2003), and can alter nutrient cycling through effects on soil bacteria and fungi, potentially costly in a nutrient-limited environment (Forbes et al. 2001). Dust in this region may be particularly harmful to organisms due to the very high content of pesticide residues and heavy metals that have resulted from drying of the Aral Sea (Micklin 2007). Strong winds are characteristic of this continental landmass, and so the dominant wind direction and subsequent dust deposition from disturbance sources have been suggested as potential drivers of vegetation response to disturbance on the Ustyurt (Micklin 2007).

In this study, we quantify habitat impacts of oil and gas extraction activities by first investigating the spatial extent of localized disturbance from infrastructure, measured in both plant species richness and vegetation cover. These two metrics together provide a broad measure of habitat condition in this region (Opp 2005), have successfully been used in previous studies investigating the impacts of disturbance (e.g. Fiori and Martin 2003; Gelbard and Harrison 2003; Simmers and Galatowitsch 2010; Lee et al. 2012), and provide a baseline for understanding the state of plant communities at a range of scales. We conducted field surveys throughout the region to determine whether: (1) there is a reduction in vegetation species richness and ground cover in disturbed relative to undisturbed (control) sites; (2) species richness and vegetation cover increase with distance from disturbance and reach background levels within 500 m from the disturbance; and (3) effects of disturbance persist over greater distances in areas downwind of the disturbance, according to dominant wind direction.

We use the results from field surveys to estimate the spatial extent of the local disturbance footprint of natural gas infrastructure across the entire plateau. By plotting the plateau-scale network of gas infrastructure, we extrapolate from localized measurements on the ground to the magnitude of the existing natural gas disturbance footprint in its entirety. This provides a measure of impact for vegetation that can inform ongoing development of a biodiversity offset policy for the region.

## Materials and Methods

### Vegetation impact transects

**Figure 1:**
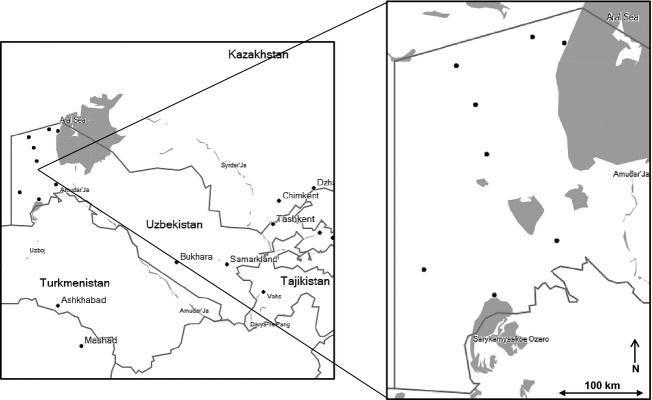
Geography of Central Asia, Uzbekistan, and the Ustyurt plateau. Circles on the detailed map are survey locations, with transects roughly equally distributed across the eight locations. Maps created using Garmin “BaseCamp”™ software.

Following a pilot expedition in September and October 2011 to test sampling protocols and arrange access, an 18-day field expedition to the Ustyurt Plateau, Uzbekistan, in May and June 2012, led by the United Nations Development Programme (UNDP), was undertaken for formal data collection. Transects employing the line-intercept method (Canfield 1941) were used to gather data on plant species richness (total number of species) and vegetation cover. Surveys were carried out at randomly selected locations within eight regions across the Ustyurt Plateau. The eight survey areas were selected by local experts to reflect the heterogeneous nature of Ustyurt biodiversity (Fig. 1; Esipov and Shomurodov 2011). Within each designated survey area, we completed as many transects as possible depending on time available, ensuring that they were spaced > 500 m apart. Transects originated at the center of components of oil and gas infrastructure and included extraction sites, pipelines, and unpaved roads known to be primarily used by oil and gas companies. For the purposes of this study, these major sources of disturbance were deemed “primary disturbances.” Transects were also undertaken at control sites without any of the above three types of infrastructure present for a minimum of 2 km. In total, 24 transects were completed: 17 disturbed sites and seven control sites.

**Figure 2:**
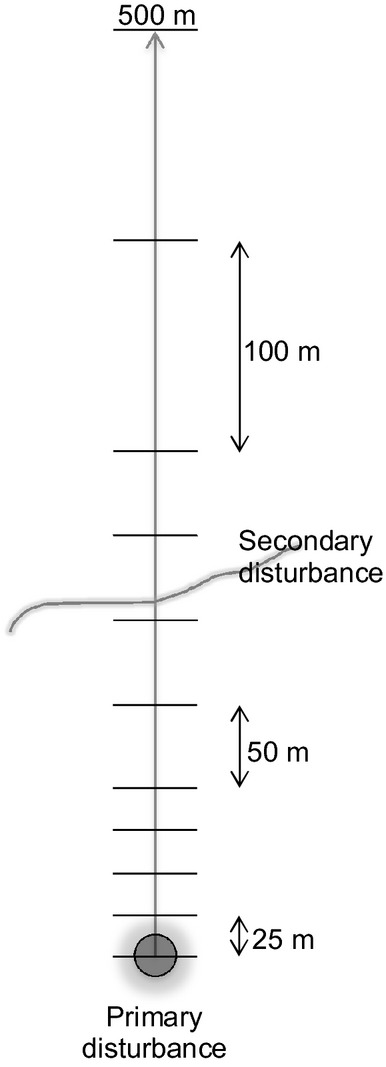
Design of spine and rib transects: the main 500-m spine transect originates from the center of disturbance with 20-m rib transects bisecting it at set intervals: 25-m intervals between 0 and 100 m (where 0 m is the center of disturbance), 50-m intervals between 100 and 300 m, and 100-m intervals between 300 and 500 m, giving increased sampling effort closer to the disturbance. The line-intercept method was used to collect species richness and vegetation cover data along each rib. Secondary disturbances (i.e. small tracks) were also recorded.

Disturbances were designated as either linear (road or pipeline) or point (“hubs” e.g. gas extraction sites). In each case, transects ran from the center of the disturbance outward for 500 m. These were perpendicular to the disturbance for linear sources and at a randomly directed angle for hubs. The size and type of each primary disturbance were recorded, and then transects were arrayed in a “spine and rib” formation, with increased sampling effort close to the disturbance source to detect any fine-scale vegetation responses (Fig. 2; Angold 1997; Lee et al. 2012). The line-intercept measurement method was applied over the 20 m length of each rib. To apply the line-intercept method, we laid down a 20 m length of rope marked with 5-cm intervals. For each plant overlaid by the rope, we recorded the length of rope intercepted by the plant when viewed from directly above. The number of different species of plant was also noted, meaning that for each rib, we could calculate both total vegetation percentage intercept (a proxy for percent cover) and species richness – species richness and percentage cover were the response variables used in analyses.

There is a network of small (< 3 m width) unpaved tracks across the Ustyurt not primarily affiliated with the extractive industry that spanned control and disturbed transects; these were labeled as secondary disturbances and noted and measured in surveys. Binary data on the presence/absence and width of secondary disturbances within 10 m and 50 m of ribs were recorded.

Due to the limited size of the dataset, we could not test the effects of each disturbance type – roads, pipelines, and extraction sites – separately, but we note that patterns of vegetation impact and recovery appeared visually similar for each disturbance type.

### Methodological considerations and compromises

It was important to ensure that sites were representative of the Ustyurt landscape as a whole, to allow scalable inferences about the impacts of infrastructure. Habitat on the Ustyurt is generally classified by local botanists into different key species associations, for example the widespread *Anabasis sp*. – *Artemisia sp*. – *Salsola sp*. association. Nevertheless, different associations are comparable in terms of both percentage cover and species diversity (Gintzburger et al. 2003; Esipov and Shomurodov 2011). Surveys were thus completed throughout the Uzbek Ustyurt and within a range of association types, but different association types were not separated in the analyses reported here.

Survey areas were chosen such that, in general, each transect remained within one association type. In order to minimize observer site selection bias as far as possible for linear disturbances, the start point was selected by walking in a fixed direction along the road/pipeline for a pre-agreed amount of time from the point at which the survey vehicle stopped (5 min), or 500 m from the end of the previous transect if more than one was being completed in a sequence. The point reached after this time was the transect start point.

### Statistical analysis

All statistical analyses were conducting using linear mixed-effects models to allow inclusion of both fixed and random effects (Bolker et al. 2009) in the “R” statistical package (R Development Core Team 2011). All models had species richness or vegetation cover as response variables. As species richness was in the form of count data, a Poisson error structure was specified for analyses. For percentage cover, data were arcsine-square root-transformed so as to follow a Gaussian rather than a binomial distribution in analyses. Maximal models included the variables site type (control vs. disturbed), distance from disturbance, disturbance width (either <3 m or >10 m), presence and width of secondary disturbances, and transect direction (as a proxy for wind direction). Wind direction data were obtained for 2009/10 from the hydrometeorological stations in Jaslyk (central Ustyurt) and in Muynak (eastern Ustyurt). As data from both sites clearly showed dominant easterly and northerly wind direction, and local wind data were not available for the transect sites visited, we assumed that this was the dominant wind direction throughout.

The transect design resulted in “ribs” being pseudoreplicated and nested within “transect.” To account for this, “transect” was fitted as a random effect within linear mixed-effects models. Maximal models including all potential explanatory variables were simplified through stepwise deletion of highest order nonsignificant terms, and model comparison using ANOVA (Crawley 2007). Models used in the analyses were as follows:

lmer(species richness ˜ control or disturbed sites × distance from disturbance + disturbance width <3 or >10 m + (1| transect), family = poisson);

and, after arcsine-square root transformation of percentage cover data:

*lmer(percentage cover ˜ control or disturbed × distance from disturbance + (1|transect)*.

*P*-values for general linear mixed-effects models involving percentage cover as the response variable were produced using Markov chain Monte Carlo (MCMC) sampling with 10,000 iterations, following Bolker et al. (2009). The package “languageR” and function “pvals.fnc” (Baayen 2011) were used with the lme4 package (Bates et al. 2012) to run MCMC and obtain *P*-values for analyses involving percentage cover data.

### Landscape footprint of oil and gas activities

Detailed official records for the spatial extent of oil and gas infrastructure on the Ustyurt were not available. Therefore, we used two methods to map the spatial distribution of key oil and gas infrastructure. First, during two field expeditions to the Ustyurt (the 2012 main expedition and the 2011 pilot expedition), a GPS track log was kept of all oil and gas infrastructure (tracks, pipelines, roads, railways, compressor stations, wells) encountered throughout the Plateau. These data were used to create an infrastructure map, extrapolated as necessary to join up known pipeline routes, and with the addition of the coordinates of additional known gas extraction facilities (UNDP 2010).

Secondly, satellite imagery was used to map all linear infrastructure on the Ustyurt Plateau clearly visible at 70 km altitude (Google Earth 2012). At this altitude, infrastructure known to be present from field observations was clearly visible, validating the method; it was assumed that other infrastructure similarly visible in the images, but not visited during the fieldwork, was also attributable to natural gas activity given the lack of other major infrastructural activities in the area. Lower altitude images, although more detailed, would have included less distinct infrastructure relating to general use of the area by herders or local traffic. It is noted that this method was not also used to map hub infrastructure: it was found difficult to distinguish with any confidence between hub gas extraction sites and other unrelated development hubs, such as settlements, takyrs (salt pans), and even water wells.

Hub disturbances consisted of the six major known gas extraction facilities: two which were visited during the fieldwork (Shakpakty and Aqsholaq) and four unnamed additional sites mapped by the UNDP (2010) but inaccessible for visitation. Limited information was available on the spatial footprint of gas extraction facilities, and those that we visited in the field could not be directly surveyed. However, a recent environmental impact assessment completed for the proposed Surgil gas development in Uzbekistan provides detailed area calculations for a major gas facility on the Ustyurt (Mott-Macdonald 2012). These calculations were taken as reflective of the spatial footprint of a standard major gas facility in the region and were used as representative data to calculate the footprints of the six hub sites. The major gas compressor plants at Jaslyk, Karakalpakia, and Kubla-na-Ustyurt were excluded, because although they represent major infrastructure, they are based within substantial settlements that would have existed in any case. Therefore, their footprint could not be attributed directly to industrial gas activities.

The physical footprint (in km^2^) occupied by each infrastructure type was calculated separately for both the field-based and satellite-based mapping techniques. In both cases, we calculated the footprint using mapped lengths with our field-measured widths of infrastructure components to obtain the overall area. The outcomes of these two different mapping approaches were compared qualitatively. Averaging the footprints obtained from both approaches produced an estimated total footprint of linear infrastructure, and the difference was treated as a simplistic measure of uncertainty. This was then summed with the total hub infrastructure footprint, to give an estimated total footprint of oil- and gas-related infrastructure on the Ustyurt.

## Results

### Vegetation impacts of infrastructure

**Figure 3:**
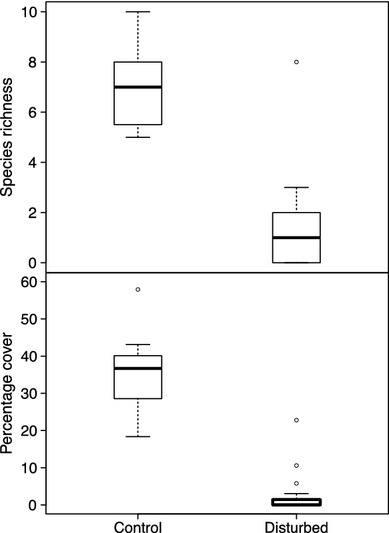
Boxplots showing differences in vegetation responses at the 0-m “rib” transect of control and disturbed sites. Differences in both species richness (*z* = −6.2, *P* < 0.001) and cover (*t* = −4.7, *P* < 0.001) are significant.

Oil- and gas-associated disturbance had a negative effect on species richness (*z* = −6.2, *P* < 0.001) and vegetation cover (*t* = −4.7, *P* < 0.001) compared with control sites (Table 1 and Fig. 3).

**Figure 4:**
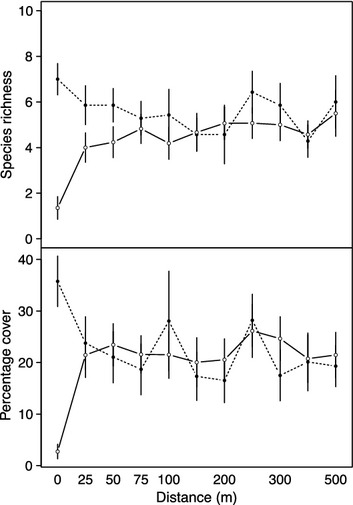
Interaction plots for richness and cover with distance. Hollow points represent disturbed sites, solid points represent controls. Graphs produced using “Sciplot” with 95% confidence intervals displayed.

Outside of actual infrastructure, species richness and cover increased to baseline levels exhibited by control sites (Fig. 4). Species richness and cover were only significantly different to baseline levels at the site of disturbance itself (0 m); at 25–500 m from disturbance, species richness and cover were not significantly different from baseline levels. Our sampling design did not anticipate complete attenuation of disturbance-associated vegetation differences within 25 m of infrastructure and was therefore not set up specifically to test for patterns between 0 and 25 m distance.

There were no significant directional impacts upon either richness or cover, as would be expected if wind deposition of dust or exhaust pollutants was an important factor for vegetation. Species richness was higher for small (< 3 m width) and large (> 10 m width) linear disturbances than it was for medium (3–10 m) linear disturbances (*z* = 2.4, *P* < 0.05 and *z* = 2.8, *P* < 0.01, respectively).

**Table 1:** Overview of vegetation response to primary disturbances. The distance at which vegetation is significantly different to baseline levels (those at the 500-m sampling point) is shown. Number of transect sites = 17, number of control sites = 7

Effect of primary disturbance		Distance at which vegetation is significantly different to baseline levels			
	
Species richness	Percentage cover	Species richness		Percentage cover	
			
Response	Response	Distance (m)	Response	Distance (m)	Response
*z* = −6.17	*t* = −4.68	0	*z* = −4.66	0	*t* = −5.2
*P* = 6.87e-10***	*P* = 0.0000***		*P* = 3.14e-6***		*P* = 0.0000***

*** Statistically significant

### Footprint of infrastructure at landscape level

**Figure 5:**
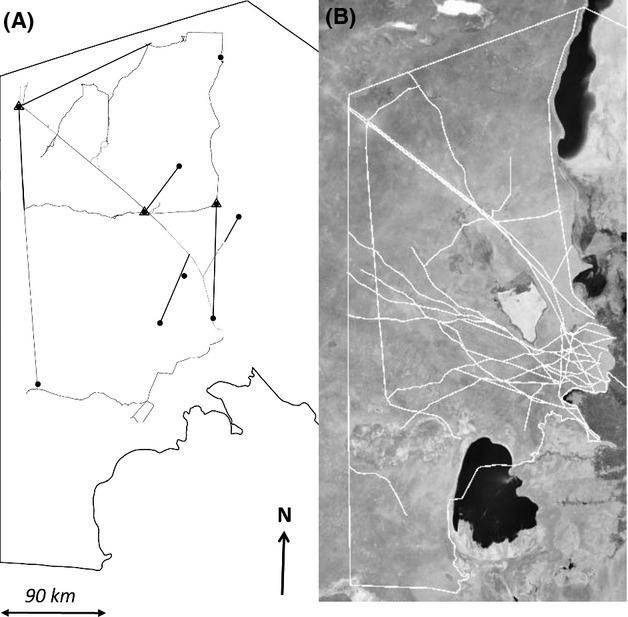
Mapping of spatial extent of gas infrastructure. (A) mapped using GPS data, where lines = roads, tracks, and pipelines, circles = settlements with gas infrastructure, triangles = known facilities used or created by the gas industry. Black lines = mapped using GPS data, gray lines = known infrastructure not mapped using GPS data; (B) linear infrastructure mapped using Google Earth (2012). White lines represent linear infrastructure – roads, railways, pipelines – associated with the oil and gas industry. Note that the large white region near the center of this figure corresponds to a dried-up saline lake.

The two mapping approaches gave visually comparable estimates of linear infrastructure presence (Fig. 5). In the NE of the plateau, infrastructure that was missed using Google Earth was recorded using GPS on the ground. This casts some doubt on the ability of the remotely sensed approach to capture all key components of infrastructure in an investigation of this type. Conversely, however, in the SE of the plateau, the Google Earth map shows linear infrastructure that was not surveyed during the expedition. Although this is simply because it was not possible to cover the plateau in its entirety during the expedition, the potential value of using the Google Earth approach to remotely map infrastructure is clear. While neither approach is comprehensive, the two arguably complement each other. The field-collected data suggested a current linear infrastructure footprint = 100 km^2^; the Google Earth method suggested a footprint = 63 km^2^.

It was not possible to survey hub extraction facilities for health and safety reasons, but we could see from visual inspection that vegetation was cleared in a similarly comprehensive manner within hub infrastructure as it was for linear infrastructure. With an assumption of immediate attenuation of disturbance effects, as conservatively documented for linear infrastructure, the footprint of extraction sites on vegetation can be calculated as the actual ground surface area occupied by extraction facilities. Extrapolating from the 23 km^2^ proposed Surgil facility (based on data provided in Mott-Macdonald 2012; Table 2), yields an estimate of a 138 km^2^ footprint.

Summing the estimates for linear and hub infrastructure, and using the difference in linear infrastructure estimates from the two different methods as a measure of uncertainty, suggests a total current footprint of 220 ± 19 km^2^. Again, this is in the context of a plateau that covers ∼ 100,000 km^2^ of Uzbekistan. Under this rough estimate, linear infrastructure constitutes 37 ± 6% of the total oil and gas infrastructure footprint.

**Table 2:** Breakdown of estimated contributions to total footprint for proposed Surgil Gas Extraction Facility (based on data in Mott-Macdonald 2012). Assumed dimensions and calculations by the authors are given in *italics* in the “Area” column. Assumed road widths, extraction radii, pipeline widths, and railway widths were based on filed observations. Values in regular font come from the report itself. Connecting (“linear”) infrastructure on site is included in the site (“hub”) footprint

Component	Subcomponent	Area (m^2^)
Main facilities		
Ustyurt Gas	Main	970,100
Chemical Complex	Storage	27,000
	Wastewater pond	240,000
Settlement	Area	850,000
	Road to settlement	5 km *by 10 m* = *50,000*
	On-site camp	? *(treated as zero)*
Gas extraction	Wells	133 wells (*radius of 170 m*) = *12,075,340*
	Gas gathering stations	6 stations (*radius of 170 m*) = *544,752*
Complex Gas Treatment Unit	Expansion of existing area	? *(treated as zero)*
Water	Water treatment plant	? *(treated as zero)*
Connecting infrastructure		
Roads	Access (UGCC)	9 ha = 90,000
	Gas gathering stations	6 roads, *3 km by 25 m* = *450,000*
	Gas wells	133 wells, average distance 4 km, *road width of 10 m* = *5,320,000*
Pipelines	Gas	115 km *by 13 m* = 1,495,000
	Gas sales	9 km *by 13 m* = 117,000
	Gas booster station	? *(treated as zero)*
	Water	27 km *by 13 m* = 351,000
Railway	Access	7 km *by 30 m* = 210,000
Electricity	Transmission line (road)	18 km *by 10 m* = 180,000
Total		
	Main facilities	14,757,192
	Connecting infrastructure	8,213,000
	Sum	22,970,192

## Discussion

### Local impact of disturbance

This study shows that natural gas infrastructure, both linear and hub, has a substantial effect on local species richness and vegetation cover in a semiarid ecosystem. But, contrary to expectation, effects are effectively limited to the footprint of physical infrastructure itself, at least in so far as our sampling scheme was equipped to measure. Future sampling that focuses on the zone between 0 and 25 meters distant from oil and gas infrastructure is key to deriving a more precise estimate. Our results did not support the idea that dust deposition significantly impacts the local cover or richness of vegetation. Future work could further quantify dust deposition including its more subtle or widespread effects (Goossens and Rajot 2008) and investigate whether directional effects on vegetation metrics vary spatially across the Ustyurt. In conjunction, these two findings suggest that the condition of vegetation was only reduced within the direct footprint of infrastructure, and there it was effectively reduced to zero: making the development of a condition-area type biodiversity offset metric, in this case, straightforward.

This study does not assess all impacts associated with oil and gas development. For instance, the industry certainly abstracts a large amount of water and may result in the keeping of additional livestock on the plateau, both of which may impact upon regional biodiversity; equally, other elements of infrastructure are thought to impact specific components of biodiversity, such as power lines causing bird mortality (Mott-Macdonald 2012). However, it does quantify the limited effects of infrastructure on vegetation as one component of a potential biodiversity offset policy in the region. Our work adds to current knowledge of disturbance effects from infrastructure on plant communities in climatically severe areas. These include Arctic tundra (Kemper and Macdonald 2009), steppe environments (Fiori and Martin 2003), and other semiarid desert regions (Simmers and Galatowitsch 2010). Oil and gas exploration is burgeoning in these habitat types; consequently, providing accurate assessments of the spatial scale of infrastructure disturbance is essential if national governments intend to ensure that any negative ecological impacts associated with the oil and gas industry are compensated for. But, as previous work strongly suggests (Benítez-López et al. 2010), the spatial scale of local disturbance is likely to extend beyond the direct physical footprint for infrastructure, when considering other components of the ecosystem such as faunal species. Hence, the area calculated here is a minimum estimate. Taking other effects of infrastructure into account is also likely to increase the relative importance of linear infrastructure in calculating overall compensation requirements, because of the known disruptive effects of anthropogenic activity on wildlife (Benítez-López et al. 2010), particularly migratory species such as the saiga antelope (e.g. Singh et al. 2010b). Further work could include investigation of the extent to which disturbance effects can be seen in other taxonomic groups on the Ustyurt such as invertebrates, reptiles, mammals, and birds, and whether the spatial scales of responses differ.

There were often observed to be relatively undisturbed “humps” between tire tracks, and there is the possibility that water pooling in tire ruts may create favorable establishment sites in arid ecosystems (Boeken and Shachak 1994; Briones et al. 1998; Brooks and Lair 2005). Furthermore, in some cases and especially for smaller, less busy roads, it is possible that roads had not been used for some time, allowing some minor colonization by vegetation. This may explain why cover and richness were not always zero within the footprint of disturbances.

### Scaling up to landscape level

Our use of mapping tools in conjunction with local-scale quantification of direct infrastructure impacts allowed estimation of the disturbance footprint from infrastructure for the whole region. This estimate is based on a number of broad assumptions and is therefore an order-of-magnitude estimate only. The two approaches we used were complementary: mapping using GPS data ground-truths infrastructure sites, while mapping using Google Earth captures all infrastructure of a certain size. Furthermore, Google Earth images present a single snapshot of the Ustyurt (without clear dates for images in the version used: Google Earth 2012), and so does not reveal the seasonality of some off-road routes. These mapping tools do, however, when used in conjunction, provide an useful estimate of the total infrastructure footprint in this region. This is likely to be a conservative estimate due to our inability to map all infrastructure associated with oil and gas activity, or to account for indirect impacts from infrastructure presence such as those upon fauna such as the saiga.

The direct footprint of the oil and gas sector is small compared with the area over which it is spread (∼ 100,000 km^2^), in fact constituting only approximately 0.2% of the plateau by area, but this does not necessarily mean that impacts are negligible on the landscape scale. Measurement of the direct footprint does not account for the fact that infrastructure may well influence the ecology of a much wider area, for instance by changing vertebrate behavior up to 5 km away from infrastructure (Benítez-López et al. 2010). The distribution of infrastructure is also important; as, beyond causing direct disturbance to wildlife, extensive linear infrastructure may physically fragment the landscape, which would particularly affect wide-ranging species (Rytwinski and Fahrig 2012) such as saigas. Quantifying these impacts is beyond the scope of this article, but these considerations are clearly important in designing biodiversity offsets, and in conservation planning for the wider landscape.

### Designing biodiversity offsets

Biodiversity offsetting has been proposed as one mechanism within a raft of initiatives for reducing negative ecological impacts associated with infrastructure development on the Ustyurt (UNDP 2010). Offsets might enable the restoration of highly degraded areas of Ustyurt habitat, commensurate with the land cleared for further infrastructure development (UNDP 2010; Bull et al. 2013b). A prerequisite for implementing offsets is the evaluation of biodiversity losses, for which some metric based on both condition and area of habitat impacted is often used (Quétier and Lavorel 2011). Our estimates of total disturbance at landscape level give a basic condition-area metric that could be used in partially quantifying the vegetation offset requirements for the region. The estimate relates to development that predates any national offset policy, and sums habitat lost over several decades, so does not constitute actual offset requirements. Furthermore, counterfactual trends in the landscape, including the influence of other industries, human settlement and agriculture, and the influence of the Aral Sea crisis (Bull et al. 2013b), would need to be considered in the design of any offset policy for the plateau. However, our approach does outline a basic methodology for calculating vegetation loss in the region and contextualizes the magnitude of potential additional losses.

More generally, our study shows that offset projects should consider linear infrastructure as well as hub development sites themselves, as linear infrastructure can constitute a large proportion of the total area impacted by developments. Finally, this investigation highlights the assumptions that must be made in calculating even one type of ecological loss for biodiversity offset schemes. In turn, this supports careful consideration of uncertainty in the development of offset policies, and the need to set up mechanisms to account for this uncertainty such as “offset multipliers” (Moilanen et al. 2009) and conservation banking (Bekessy et al. 2010). In the development of offset policy, it can often be assumed that the uncertainty dealt with by multipliers and biodiversity banking is that associated with the offset action (the biodiversity gain from the offset), and not the quantification of the original impact (e.g. Defra 2011). But Moilanen et al. (2009) and Bekessy et al. (2010) suggest instead that multipliers include the uncertainty around the damage incurred, that is, the conservation value of land that is developed, and that biodiversity banking should recognize uncertainty in impacts as well as actions. Considering uncertainty in the quantification of residual development impacts on biodiversity, as is carried out in our preliminary study here, is consequently crucial for the implementation of offset actions.

## References

[b1] Angold PG (1997). The impact of a road upon adjacent heathland vegetation: effects on plant species composition. J. Appl. Ecol.

[b2] Baayen RH (2011). http://cran.r-project.org/package=languageR.

[b3] Baillie JEM, Hilton-Taylor C, Stuart SN (2004). 2004 IUCN red list of threatened species. A global species assessment.

[b4] Bates D, Maechler M, Bolker B (2012). http://cran.r-project.org/package=lme4.

[b5] Bekessy SA, Wintle BA, Lindenmayer DB, Mccarthy MA, Colyvan M, Burgman MA (2010). The biodiversity bank cannot be a lending bank. Conserv. Lett.

[b6] Benítez-López A, Alkemade R, Verweij PA (2010). The impacts of roads and other infrastructure on mammal and bird populations: a meta-analysis. Biol. Conserv.

[b7] Boeken B, Shachak M (1994). Desert plant communities in human-made patches–implications for management. Ecol. Appl.

[b8] Bolker BM, Brooks ME, Clark CJ, Geange SW, Poulsen JR, Stevens HH (2009). Generalized linear mixed models: a practical guide for ecology and evolution. Trends Ecol. Evol.

[b9] Briones O, Montana C, Ezcurra E (1998). Competition intensity as a function of resource availability in a semiarid ecosystem. Oecologia.

[b10] Brooks ML, Lair B, R. H Webb, L Fenstermaker, J Heaton, D Hughson (2005). Ecological effects of vehicular routes in a desert ecosystem.

[b11] Brown G, Schoknecht N (2001). Off-road vehicles and vegetation patterning in a degraded desert ecosystem in Kuwait. J. Arid Environ.

[b12] Bull JW, Suttle KB, Gordon A, Singh NJ, Milner-Gulland EJ (2013a). Biodiversity offsets in theory and practice. Oryx.

[b13] Bull JW, Suttle KB, Singh NJ, Milner-Gulland EJ (2013b). Conservation when nothing stands still: moving targets and biodiversity offsets. Front. Ecol. Environ.

[b14] Canfield RH (1941). Application of the line interception method in sampling range vegetation. J. Forest.

[b15] Coffin AW (2007). From roadkill to road ecology: a review of the ecological effects of roads. J. Transp. Geogr.

[b16] Crawley MJ (2007). The R book.

[b17] Cui B, Zhao S, Zhang K, Li S, Dong S, Bai J (2009). Disturbance of Dabao highway construction on plant species and soil nutrients in Longitudinal Range Gorge Region (LRGR) of Southwestern China. Environ. Monit. Assess.

[b18] Curatolo JA, Murphy SM (1986). The effects of pipelines, roads, and traffic on the movements of Caribou, Rangifer tarandus. Can. Field-Nat.

[b19] Defra (UK Department for the Environment, Food, and Rural Affairs) (2011). Technical paper: proposed metric for the biodiversity offsetting pilot in England.

[b20] Dorian JP (2006). Central Asia: a major emerging energy player in the 21st century. Energy Policy.

[b21] Dyer SJ, O'Neill JP, Wasel SM, Boutin S (2002). Quantifying barrier effects of roads and seismic lines on movements of female woodland caribou in northeastern Alberta. Can. J. Zool.

[b22] E&P Forum/UNEP (1997). Environmental management in oil and gas exploration and production: an overview of issues and approaches.

[b23] Effimoff I (2000). The oil and gas resource base of the Caspian region. J. Petrol. Sci. Eng.

[b24] EIA (2012). http://www.eia.gov/countries/cab.cfm?fips=UZ.

[b25] Epstein PR, Selber J (2002). Oil: a life cycle analysis of its health and environmental impacts.

[b26] Esipov AV, Shomurodov H (2011).

[b27] Farmer A (1993). The effects of dust on vegetation - a review. Environ. Pollut.

[b28] Fiori SM, Martin S (2003). Potential impacts of petroleum exploration and exploitation on biodiversity in a Patagonian Nature Reserve, Argentina. Biodivers. Conserv.

[b29] Forbes BC, Ebersole JJ, Strandberg B (2001). Anthropogenic disturbance and patch dynamics in circumpolar arctic ecosystems. Conserv. Biol.

[b30] Forman RTT, Alexander LE (1998). Roads and their major ecological effects. Annu. Rev. Ecol. Evol. Syst.

[b31] Gadsdon SR, Power SA (2009). Quantifying local traffic contributions to NO_2_ and NH_3_ concentrations in natural habitats. Environ. Pollut.

[b32] Gelbard JL, Belnap J (2003). Roads as conduits for exotic plant invasions in a semiarid landscape. Conserv. Biol.

[b33] Gelbard JL, Harrison S (2003). Roadless habitats as refuges for native grasslands: interactions with soil, aspect, and grazing. Ecol. Appl.

[b34] Gintzburger G, Toderich KN, Mardonov BK, Mahmudov MM (2003). Rangelands of the arid and semi-arid zones in Uzbekistan.

[b35] Gintzburger G, Rajabov T, Bull J (2011).

[b36] Google Earth 6.0 (2012). http://www.google.com/earth/index.html.

[b37] Goossens D, Rajot JL (2008). Techniques to measure the dry aeolian deposition of dust in arid and semi-arid landscapes: a comparative study in West Niger. Earth Surf. Proc. Land.

[b38] Grantz DA, Garner JHB, Johnson DW (2003). Ecological effects of particulate matter. Environ. Int.

[b39] ten Kate K, Bishop J, Bayon R (2004). Biodiversity offsets: views, experience, and the business case.

[b40] Kemper TJ, Macdonald ES (2009). Directional change in upland tundra plant communities 20-30 years after seismic exploration in the Canadian low-arctic. J. Veg. Sci.

[b42] Kumpula T, Pajunen A, Kaarlejarvi E, Forbes BC, Stammler F (2011). Land use and land cover change in Arctic Russia: ecological and social implications of industrial development. Glob. Environ. Change.

[b43] Lee MA, Davies L, Power SA (2012). Effects of roads on adjacent plant community composition and ecosystem function: an example from three calcareous ecosystems. Environ. Pollut.

[b44] Lovich J, Bainbridge D (1999). Anthropogenic degradation of the Southern California desert ecosystem and Prospects for natural recovery and restoration. Environ. Manage.

[b46] Mallon DP (2008). http://www.iucnredlist.org.

[b47] Micklin P (2007). The Aral sea disaster. Annu. Rev. Earth Planet. Sci.

[b48] Millennium Ecosystem Assessment (2005). Ecosystems and human well-being: biodiversity synthesis.

[b49] Moilanen A, Teeffelen JA, Ben-Haim Y, Ferrier S (2009). How much compensation is enough? A framework for incorporating uncertainty and time discounting when calculating offset ratios for impacted habitat. Restor. Ecol.

[b50] Mott-Macdonald (2012). http://www.agaportal.de/pdf/nachhaltigkeit/eia/eia_usbekistan_chemie2.pdf.

[b51] OGP/IPIECA (2011). Ecosystem services guidance: biodiversity and ecosystem services guide and checklists.

[b52] Opp C (2005). Desertification in Uzbekistan. Geogr. Rundsch. Int. Ed.

[b53] Quétier F, Lavorel S (2011). Assessing ecological equivalence in biodiversity offset schemes: key issues and solutions. Biol. Conserv.

[b54] Quintero J, Mathur A (2011). Biodiversity offsets and infrastructure. Conserv. Biol.

[b55] R Development Core Team (2011). http://www.r-project.org/.

[b56] Rabanal LI, Kuehl S, Mundry R, Robbins MM, Boesch C (2010). Oil prospecting and its impact on large rainforest mammals in Loango National Park, Gabon. Biol. Conserv.

[b57] Rapport DJ, Whitford WG (1999). How ecosystems respond to stress: common properties of arid and aquatic systems. Bioscience.

[b58] Rytwinski T, Fahrig L (2012). Do life history traits explain population responses to roads? A meta-analysis. Biol. Conserv.

[b59] Simmers SM, Galatowitsch SM (2010). Factors affecting revegetation of oil field access roads in semiarid grassland. Restor. Ecol.

[b60] Singh NJ, Grachev IA, Bekenov AB, Milner-Gulland EJ (2010a). Tracking greenery across a latitudinal gradient in central Asia - the migration of the saiga antelope. Divers. Distrib.

[b61] Singh NJ, Grachev IA, Bekenov AB, Milner-Gulland EJ (2010b). Saiga antelope calving site selection is increasingly driven by human disturbance. Biol. Conserv.

[b62] Thibault M, Blaney S (2003). The oil industry as an underlying factor in the bushmeat crisis in central Africa. Conserv. Biol.

[b63] Trombulak SC, Frissell CA (2000). Review of ecological effects of roads on terrestrial and aquatic communities. Conserv. Biol.

[b64] UNDP (2010). http://www.undp.uz/en/projects/project.php?id=166.

[b65] USAID (2001). Biodiversity assessment for Uzbekistan.

[b66] Vistnes I, Nellemann C (2007). The matter of spatial and temporal scales: a review of reindeer and caribou response to human activity. Polar Biol.

[b67] Walker BL, Naugle DE, Doherty KE (2007). Greater sage-grouse population response to energy development and habitat loss. J. Wildl. Manage.

[b68] White RP, Nackoney J (2003). Drylands, people, and ecosystem goods and services: a web-based geospatial analysis.

[b69] Zeng SL, Zhang TT, Gao Y, Ouyang ZT, Chen JK, Li B (2011). Effects of road age and distance on plant biodiversity: a case study in the Yellow River Delta of China. Plant Ecol.

